# Supplementary figures and images for: Enhancing clinical decision-making: Sysmex UF-5000 as a screening tool for bacterial urinary tract infection in children
Source: PLoS One. 2024 Jun 12;19(6):e0304286. doi: 10.1371/journal.pone.0304286 (PMC11168643; doi:10.1371/journal.pone.0304286)

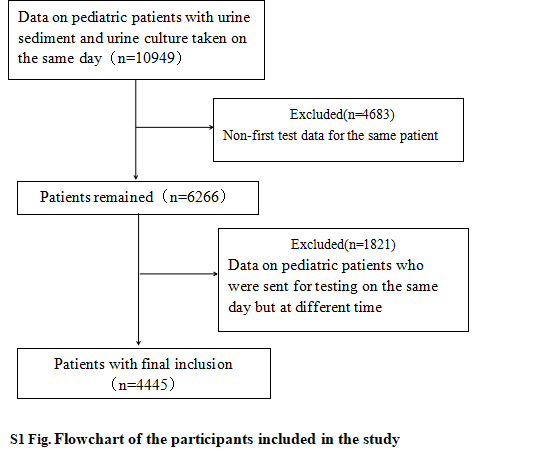

Supplement: S1 Fig — (TIF) [file pone.0304286.s001.tif]
