# Supplementary material for: Enhancing clinical decision-making: Sysmex UF-5000 as a screening tool for bacterial urinary tract infection in children
Source: PLoS One. 2024 Jun 12;19(6):e0304286. doi: 10.1371/journal.pone.0304286 (PMC11168643; doi:10.1371/journal.pone.0304286)
Supplement: Attachment — Submitted filename: Comments for PONE-D-24-04363.docx [file pone.0304286.s002.docx]

This study investigated the performance of Sysmex UF-5000 analyzer parameters for screening urinary tract infection (UTI) in children. I believe the manuscript should be rejected due to the following reasons:

1、 The manuscript is poorly organized and its readability needs to be extensively improved.
2、 The language quality does not meet the publication criteria of PLoS ONE.
3、 The authors should provide more details about the inclusion and exclusion criteria for the patient enrollment.
4、 I suggest the authors use a flowchart to depict participant selection.
5、 How to handle duplicated participants?
6、 The type of data collection (retrospective or prospective) should be reported.
7、 The sample sizes of UTI and non-UTI were not reported.
8、 In table 2, the rate of missed diagnosis should be reported.
9、 The novelty and strength of this study were not well discussed in the discussion section.

Minor comments

1、The upper limit of AUC’s confidence interval should not exceed 1.

2、The 95% confidence intervals for sensitivity and specificity should be reported
