# Supplementary material for: Enhancing clinical decision-making: Sysmex UF-5000 as a screening tool for bacterial urinary tract infection in children
Source: PLoS One. 2024 Jun 12;19(6):e0304286. doi: 10.1371/journal.pone.0304286 (PMC11168643; doi:10.1371/journal.pone.0304286)
Supplement: Attachment — Submitted filename: Response to Reviewers.docx [file pone.0304286.s003.docx]

Dear Editor and Reviewers,

Thank you for your email and for the reviewers’ comments concerning our manuscript entitled “Enhancing clinical decision-making: Sysmex UF-5000 as a screening tool for bacterial urinary tract infections in children” **( PONE-D-24-04363).** The comments are all valuable and very helpful for revising and improving our paper and have important guiding significance for our research. We have read the comments carefully and have made corrections which we hope meet with approval. The revised portions are mark by “Track Change” in red font throughout the revised manuscript. The main corrections in the paper and the response to the editor and reviewer comments are as follows.

**Editorial Office Comments：**

**1.** **Please ensure that your manuscript meets PLOS ONE's style requirements, including those for file naming. The PLOS ONE style templates can be found at 
https://journals.plos.org/plosone/s/file?id=wjVg/PLOSOne_formatting_sample_main_body.pdf and** [**https://journals.plos.org/plosone/s/file?id=ba62/PLOSOne**](https://journals.plos.org/plosone/s/file?id=ba62/PLOSOne)

**_formatting_sample_title_authors_affiliations.pdf**

**Reply:** Dear editor, we have revised according to the style requirements of PLOS ONE. The revised portions are mark by “Track Change” in red font throughout the revised manuscript.

**2. We suggest you thoroughly copyedit your manuscript for language usage, spelling, and grammar. If you do not know anyone who can help you do this, you may wish to consider employing a professional scientific editing service.**

**Reply:** Dear editor, we have employing a professional scientific editing service (Editage) to help us language editing.

**3. For studies involving third-party data, we encourage authors to share any data specific to their analyses that they can legally distribute. PLOS recognizes, however, that authors may be using third-party data they do not have the rights to share. When third-party data cannot be publicly shared, authors must provide all information necessary for interested researchers to apply to gain access to the data.(https://journals.plos.org/plosone/s/data-availability#loc-acceptable-data-access-restrictions)**

**Reply:** **Data Availability:** Data cannot be shared publicly because of hospital data privacy. Data are available from the Children's Hospital Affiliated to Shandong University Ethics Committee (contact via 008653159616082) for researchers who meet the criteria for access to confidential data.

**4. While revising your submission, please upload your figure files to the Preflight Analysis and Conversion Engine (PACE) digital diagnostic tool,** [**https://pacev2.apexcovantage.com/**](https://pacev2.apexcovantage.com/)**. PACE helps ensure that figures meet PLOS requirements.**

**Reply:** Dear editor, following your suggestion, we modified the figure accordingly.

**Reviewers' comments:**

**Reviewer #1**

**1. Conclusions: I like that the authors write Gram usually with capital letter, because it is the name of a person. In Conclusion it should also be written with capital letter.**

**Reply:** We greatly appreciate the reviewer’s comments. Following the reviewer’s suggestion, in the conclusion, the word “gram “was replaced with “Gram”.

**2. Urine culture and identification of microbes. “If there was sterile growth”. Although the authors used 10 µl for urine culture, also in case of no growth, one cannot say it is sterile. Therefore, better: “If there was no growth”.**

**Reply:** We greatly appreciate the reviewer’s comments. Following the reviewer’s suggestion, we use " If there was no growth " instead of " If there was sterile growth " in the revised manuscript.

**3.** **Table 2. Typing error: Tocal? Maybe should read Total. It was used 3 times.
Otherwise I have no further comments.**

**Reply:** Thank you for pointing out these errors. We have made the necessary corrections and conducted a thorough review of the spelling in the document.

**Reviewer #2**

**1. Abstract (methods): “We analyzed 4445 patient information of children….” This sentence should be rewritten in a better and more informative way.**

**Reply:** We sincerely appreciate the reviewer's feedback and have revised this section based on the suggestions provided. The revised portions are indicated by 'Track Changes' in red font in the updated version of our manuscript.

**2. Ethics statement: “And began accessing data on December 29, 2023. The author had access to information that could identify individual participants during data collection…” This part should be rewritten in a better English.**

**Reply:** We sincerely appreciate the reviewer's feedback and have revised this section based on the suggestions provided. The revised sections are highlighted with 'Track Changes' in red font in the revised version of our manuscript.

**3. Results/Microbial species: *“C. tropical”* should be spelled out to *“Candida tropicalis”***

**Reply:** We greatly appreciate the reviewer’s comments. In accordance with the reviewer's recommendation, we have updated the terminology in our manuscript to use “*Candida tropicalis*” instead of “*C. tropical*”.

**4. Table 1: “*Proteus mirabili*” should read *“Proteus mirabilis”***

**Reply:** We greatly appreciate the reviewer’s comments. Following the reviewer’s suggestion, we use " *Proteus mirabilis* " instead of " “*Proteus mirabili* " in the revised manuscript.

**5. Results/Diagnostic performance of UF-5000: “Excluding samples from the fungus culture and the < 104 CFU/mL group, an ROC curve…” should read “…, a ROC curve…”**

**Reply:** Thank you for pointing this out. We have corrected these grammar mistakes mentioned above and have carefully reviewed the language throughout the document. **6. The “limitations” should be mentioned before the conclusion.**

**Reply:** We greatly appreciate the reviewer’s comments and have adjusted the order of these two parts as suggested.

**Reviewer #3**

**1.Abstract, Results. The author mentions that unnecessary culture could be avoided in 28.1%. Are there a reason for presented the numbers with 3 digits? Why not say 28%. I hardly think the estimates is that precise?**

**Reply:** We appreciate the reviewer's feedback on our work. In order to minimize false negatives, a higher sensitivity of 99% was applied as a condition. The unnecessary cultures percentage was calculated as True Negative divided by the Total Number, resulting in 0.280839231547 (1111/3956). The information is presented in Table 2, where we have rounded the numbers to one decimal place to highlight subtle differences in the data. This approach was particularly important for metrics like negative predictive value (NPV) %. Therefore, we ensured consistency by consistently using one decimal place throughout the entire analysis process.

**Table 2. Performance of UF-5000 with various cut-off values for BACT counts in 3956 samples.**

| Performance | Cut-off BACT Count(/μL) | | | |
| --- | --- | --- | --- | --- |
|  | 92.5 | 47.5 | 13.5 | 7.8 |
| Sensitivity % | 85.3 | 90.0 | 95.0 | 99.0 |
| Specificity % | 91.5 | 85.6 | 66.4 | 32.3 |
| Negative Predictive value(NPV) % | 97.7 | 98.3 | 99.0 | 99.6 |
| Positive Predictive value(PPV) % | 59.8 | 48.3 | 29.8 | 17.9 |
| False negative rate % | 14.6 | 9.7 | 4.5 | 0.9 |
| Unnecessary cultures % | 79.6 | 74.5 | 57.8 | 28.1 |
| True Positives(n) | 438 | 463 | 490 | 508 |
| False positives(n) | 294 | 495 | 1156 | 2332 |
| True negative(n) | 3149 | 2948 | 2287 | 1111 |
| False negatives(n) | 75 | 50 | 23 | 5 |

**2.Abstract, Conclusion. Cultures screened with UF-5000 showed superior results in identifying…superior in comparison to what? I don’t think you mean compared to urine culture? Or do you mean compared to previous flow cytometry models like UF-1000 or compared to Urine stricks? The UF-5000 may be faster than culture but not better. It may also be better that other fast screening methods.
Reply:** The reviewer's feedback is greatly appreciated. Our research found that cultures screened with UF-5000 demonstrated superior results in identifying negative cultures. Specifically, the consistency between UF-5000 BACT-Info flags indicating 'Unclassified' and negative urine cultures was 97.9% (3370 / 3443) (highlighted in yellow in Table 3). Moreover, Cultures screened with UF-5000 showed superior results in identifying significant bacterial growth, Specifically, For samples with ≥105 CFU/mL bacteria, “Gram-negative?” UF-5000 BACT-Info flags showed a better concordance of 74.1% (60/81) compared to 104-105 CFU/mL bacteria, with 57.5% (142/257) (highlighted in green in Table 3).Similarly, 53.4% (16/30) of urine cultures with growth ≥105CFU/ml of GP bacteria showed higher concordance than urine cultures with 104–105CFU/ml 34.8% (48/138) (highlighted in blue in Table 3). This sentence is indeed ambiguous, we appreciate the feedback provided and have revised the expression in the section accordingly, the revised portions are mark by “Track Change” in red font in the updated version of our manuscript.

**Table 3. Comparison of the UF-5000 BACT-Info flags and urine culture results**

| BACT-Info flag by UF-5000 | Urine Culture | | | |
| --- | --- | --- | --- | --- |
|  | Gram-negative bacterial growth(n,%) | Gram-positive bacterial growth  (n,%) | Gram-negative and  positive mixed growth  (n,%) | No growth  (n,%) |
| Total specikens |  |  |  |  |
| Total | 328 | 168 | 17 | 3443 |
| Gram-negative? | 202(61.6) | 6(3.6) | 4(23.5) | 33(0.9) |
| Gram-positive? | 5(1.5) | 64(38.1) | 3(17.6) | 20(0.6) |
| Gram-pos/neg? | 41(12.5) | 13(7.7) | 2(11.8) | 20(0.6) |
| Unclassified | 80(24.4) | 85(50.6) | 8(47.1) | 3370(97.9) |
| ≥10^5^CFU/ml |  |  |  |  |
| Total | 81 | 30 | 2 | NA |
| Gram-negative? | 60(74.1) | 4(13.3) | 0 | NA |
| Gram-positive? | 0 | 16(53.4) | 0 | NA |
| Gram-pos/neg? | 10(12.3) | 3(10.0) | 1(50.0) | NA |
| Unclassified | 11(13.6) | 7(23.3) | 1(50.0) | NA |
| 10^4^-10^5^ CFU/ml |  |  |  |  |
| Total | 247 | 138 | 15 | NA |
| Gram-negative? | 142(57.5) | 2(1.4) | 4(26.7) | NA |
| Gram-positive? | 5(2.0) | 48(34.8) | 3(20.0) | NA |
| Gram-pos/neg? | 31(12.6) | 10(7.2) | 1(6.7) | NA |
| Unclassified | 69(27.9) | 78(56.6) | 7(46.6) | NA |

**3.** **Introduction/discussion. The authors mention that** **rapid screening test for UTI**

**is needed for children.** **But there is nothing mentioned about urine dipsticks that are ruinously used in many settings for rapid screening, including in children >2 years of age. Is that because the dipsticks are not used at this children’s’ hospital? It may be worth mentioning that there are a lot of rapid screen test on the marked, but they do show inadequate performance compared to culture.
Reply:** Our hospital predominantly utilizes the UC-3500 urine dry chemical analyzer for testing with dipsticks. This study focuses on exploring the new parameter of bacterial information provided by the UF-5000, and reduce unnecessary cultures based on bacterial count. We appreciate the reviewer for providing valuable feedback, which serves as a helpful reminder for our future research endeavors. In our next investigation, we plan to delve deeper into the joint testing of UC-3500 and UF-5000. As pointed out by the reviewer, the urine dipstick serves as a rapid screening test, and we have incorporated comments on its application for UTIs in the introduction of our revised manuscript.

I concur with the reviewer's observation that the UF-5000 may be faster than culture but not better. Urine culture remains the gold standard for diagnosing UTIs。 and these contents are also reflected in the discussion. **4.Urine Culture: Inoculation involved using a calibrated loop to apply 10 µL culture. It is common to use a 1-μL inoculating loop, so can you elaborate on the choice of the 10 µL loop? It may be a good point to include in your discussion as many of the studies you compare uses 1 µL loop cultures and therefore you may not compare to the same culture standard.
Reply:** We greatly appreciate the reviewer’s comments. Based on the health industry standard WS/T489-2016 of the People's Republic of China regarding 'Laboratory Diagnosis of Urinary Tract Infections', section 8.4.1 on Selection of Culture Types and section 10 on Interpretation of Results, it is recommended to use either a 1μL or 10μL inoculating loop. However, our hospital currently does not have the 1μL loop and therefore we typically use the 10µL loop. It is worth noting that while using the 10µL loop does not impact the judgment criteria. Furthermore, the 10μL loop can be effectively reused after proper sterilization with an alcohol lamp, offering a cost-saving measure. Please refer to the industry standard guidelines for more detailed information.

8.4.1 Selection of Cultivation Types

The clean midstream urine culture method involved inoculating 1μL or 10μL onto blood agar plates, MacConkey or Chinese blue agar plates. All plates were incubated for 18-24h at 37 °C in 5% CO_2_. If there was bacterial growth, the colonies were counted. If there was no growth, cultivation continued for 48h.

10 Result interpretation

10.1 Summary: With a 1μL inoculum, the count results in plate colony count×10^3^ CFU/mL. With a 10μL inoculum, the count results in plate colony count×10^2^CFU/mL. Clinical physicians are advised to analyze the clinical significance of urine culture results in conjunction with urine routine results.

10.2 General explanation: Following quantitative cultivation of clean midstream urine, a single bacterial colony count ≥ 10^5^CFU/mL may indicate infection, while <10^4^CFU/mL may be contamination. The range of 10^4^CFU/mL to 10^5^CFU/mL requires evaluation based on the patient's clinical manifestations. These parameters are usually sufficient for accurate diagnosis of most cases of pyelonephritis and cystitis.

In conclusion, while various specifications of inoculating loop are employed, the judgment criteria remain consistent post-conversion.

**5.** **Urine culture. BACT counts ≥ 10^4^CFU/mL should be changed to BACT counts ≥ 10^4^ CFU/mL. Do the sentence BACT counts ≥ 10^4^CFU/mL (when symptomatic) where considered positive, mean that samples with ≥ 10^4^CFU/mL where excluded if the patient did not have any symptoms? I don’t think you can exclude culture positive samples from the dataset, at least not without mentioning this an exclusion criteria. Also how where ≥ 10^3^CFU/mL classified? As negative? You may stick to the recommended cut-off urine cultures in children at ≥ 104 CFU/mL.: 1999. Practice parameter: the diagnosis, treatment, and evaluation of the initial urinary tract infection in febrile infants and young children. American Academy of Pediatrics. Committee on Quality Improvement. Subcommittee on Urinary Tract Infection. Pediatrics 103:843-852
Reply:** Culture positive samples were not excluded from the dataset, and all children with BACT > 10^4^ CFU/mL were included in the research. We acknowledge the reviewer's feedback and have made the required revisions, as well as cited relevant articles in our manuscript. The revised sections are highlighted with 'Track Changes' in red font on pages 5-6 of the original text. Following industry standards where ≥ 10^3^ CFU/mL is considered contamination, data with BACT < 10^4^ CFU/mL were not included in the results of the second to fourth parts. **6. Results UF-5000 BACT and WBC count and Figure 1. You mentioned in caption to fig.1 that fungi are excluded. Why is that? Are fungi defined as culture negative or positive? You need to define this or explain the rationale about excluding them. Fungi is a non-bacterial UTI.
Reply:** We appreciate the valuable feedback provided by the reviewer. It is important to note that fungi are considered a non-bacterial UTI, with the corresponding parameter in the Sysmex UF-5000 being YSL counts. While fungi are typically identified through culture positivity, our research parameters, specifically BACT counts and BACT-Info, primarily focus on bacterial UTIs. Additionally, the percentage of fungal samples in our study was relatively low at 1.2% (54 out of 4445 samples). Therefore, we made the decision to exclude fungal data in order to maintain the scientific integrity of our analysis. At present, we have not conducted a correlation study between fungal UTIs and YSL counts parameter due to the limited volume of available data. In order to express our research content more clearly, we have made modifications to the title：Enhancing clinical decision-making: Sysmex UF-5000 as a screening tool for bacterial urinary tract infections in children.

**7. You identified 513 samples with significant growth. I think you should use the same term in the manuscript for culture positive. So may change the sentence to 513 culture positive samples.
Reply:** We greatly appreciate the reviewer’s comments. Following the reviewer’s suggestion, we use " 513 culture positive samples " instead of " 513 samples with significant growth. " in the revised manuscript.

**8. Diagnostic performance of UF-5000. Again rationale for excluding samples with fungi.**

**Reply:** We appreciate the reviewer's comments and have incorporated corresponding explanations in question 6.

**Reviewer #4**

**1.The manuscript is poorly organized and its readability needs to be extensively improved.**

**Reply:** Thank you for your feedback. We have thoroughly revised the manuscript based on the reviewer's comments. We believe that our response and revisions address the concerns raised and enhance the quality of the publication. We hope that the revised version meets the standards for publication. The revised sections are mark by “Track Change” in red font throughout the revised manuscript.

**2.The language quality does not meet the publication criteria of PLoS ONE.**

**Reply:** Thank you for your feedback. we have employing a professional scientific editing service to help us language editing.
**3.The authors should provide more details about the inclusion and exclusion criteria for the patient enrollment.**

**Reply:** This retrospective study utilized data extracted from medical records. Urine culture was employed as the reference standard for the research. To assess the performance of Sysmex UF-5000, it was imperative to ensure that the detection outcomes from both methods were derived from the same urine sample for comparability. Initially, data was gathered from pediatric patients who underwent urine sediment and urine culture tests on the same day. Subsequently, only the initial test results for each patient were retained based on their ID. Patients who underwent examinations on the same day but at different times, determined by the specific time of collection, were then excluded from the analysis. Finally, 4445 childrens were included in the study.

**4.I suggest the authors use a flowchart to depict participant selection.**

**Reply:** We greatly appreciate the reviewer’s comments. Based on the reviewer's suggestion, we have incorporated a flowchart in the supporting information to describe the selection of participants.

**S1 Fig.** Flowchart of the participants included in the study

Data on pediatric patients with urine sediment and urine culture taken on the same day（n=10949）

Excluded(n=4683)

Non-first test data for the same patient

Patients remained（n=6266）

Excluded(n=1821)

Data on pediatric patients who were sent for testing on the same day but at different time

Patients with final inclusion（n=4445）

**5.How to handle duplicated participants?**

**Reply:** we will only retain the first test data for each patient based on their unique patient ID.

**6.The type of data collection (retrospective or prospective) should be reported.**

**Reply**: As a retrospective study, this detail has been included in the methodology section.

**7.The sample sizes of** **UTI and non-UTI were not reported.**

**Reply:** Thank you for your feedback. Based on the health industry standard WS/T489-2016 of the People's Republic of China regarding 'Laboratory Diagnosis of Urinary Tract Infections' and reference[14]：BACT counts ≥ 10^4^ CFU/mL were considered culture positive, BACT counts < 10^4^CFU/mL raised suspicion of contamination [14]. The urine was classified as contaminated if three or more colonies without a predominant organism were observed, representing the diagnostic standard for pediatric patients in the author's institution. We divided 4445 children into the following groups:

Patients with final inclusion（n=4445）

Fungal（n=54）

≥10^5^ CFU/mL（n=113）

No growth

（n=3443）

<10^4^ CFU/mL（n=435）

10^4^-10^5^ CFU/mL（n=400）

**UTIs**

（n=567）

**Non-UTIs**（n=3878）

This study aims to validate the performance and intended use of the novel UF-5000 fluorescence flow cytometer. The findings will be compared to culture results to evaluate the accuracy of the UF-5000 in discriminating between gram-positive and gram-negative bacteria. Therefore, research results were not grouped according to UTI and non-UTI categories, but instead, utilizing grouped data for analysis can provide a clearer demonstration of our research results.

**8.In table 2, the rate of missed diagnosis should be reported.**

**Reply:** According to the reviewer's feedback, we have added the missed diagnosis rate, which is the false negative rate in Table 2.
**9.The novelty and strength of this study were not well discussed in the discussion section.**

**Reply:** We greatly appreciate the reviewer’s comments. The Sysmex UF-5000, a third-generation fully automated urine flow cytometer, a new function is its ability to distinguish gram-positive and gram-negative bacteria in urine samples. The majority of previous studies have focused on adult samples. To this end, we analyzed 4445 patient information of children and compared UF-5000 results with urine cultures. It was the most significant number of analyzed urine samples among the UF-5000 studies, and evaluated for the first time the performance of the Sysmex UF-5000 flow cytometer as a screening tool for UTIs in children, and its ability to differentiate Gram typing of bacteria in UTI samples using new parameters, and these contents are also reflected in the discussion.

**Minor comments
1.The upper limit of AUC’s confidence interval should not exceed 1.**

**Reply:** Thank you for pointing this out. In the original text, we utilized the calculation results from GraphPad Prism (Fig 1). However, in response to the reviewer's feedback, we reanalyzed the data using SPSS (Fig 2). We had not previously noted this discrepancy, while the AUC, SP, and SE values from both software are consistent (Fig 3), there are differences in the 95% confidence intervals.

**2.The 95% confidence intervals for sensitivity and specificity should be reported**

**Reply:** we appreciate the feedback provided and have revised the expression in the section accordingly:

**Reviewer #5**

**1.Introduction
- Improper collection is more likely to lead to contamination and hence false positive results
Reply:** We greatly appreciate the reviewer’s comments. Following the reviewer’s suggestion, the statements mentioned above have been revised in our manuscript accordingly.

**- Comment on the use of dipstick test for UTIs in children (given that it also provides rapid results and is very cheap)**

**Reply:** We greatly appreciate the reviewer’s comments. Following the reviewer’s suggestion, we have incorporated comments on dipstick test application for UTIs in the introduction of our revised manuscript. The revised sections are highlighted with 'Track Changes' in red font on pages 4 of the original text.

**- When introducing Sysmex, please add the manufacturer and location**

**Reply:** We greatly appreciate the reviewer’s comments. Following the reviewer’s suggestion, we have added the manufacturer and location.

**- Explain to the reader by which mechanism can the Sysmex differentiate between Gram positive and Gram negative organisms**

**Reply:** We greatly appreciate the reviewer’s comments. The mechanism by which Sysmex distinguishes between Gram positive and Gram negative bacteria is as follows：Gram-negative and Gram-positive bacteria are differentiated by difference in the thickness of the peptidoglycan layer in their cell walls and the levels of transferable pigments. Gram-positive bacteria have a thicker peptidoglycan layer, leading to stronger forward scattered light intensity (FSC) and less pigment penetration, resulting in lower lateral fluorescence intensity (FL). Conversely, Gram-negative bacteria have a thinner peptidoglycan layer, producing weaker FSC and allowing more pigment penetration, resulting in higher FL.  As a result, Gram-positive bacteria tend to be located in regions with larger angles on scatter plots, while Gram-negative bacteria are typically found in flatter areas with smaller angles.

And according to the suggestions of the reviewers, the revised sections are highlighted with 'Track Changes' in red font on pages 4 of the original text.

**2.Methods
- “If there is sterile growth” please rephrase to no growth or similar**

**Reply:** We greatly appreciate the reviewer’s comments. Following the reviewer’s suggestion, we use " If there was no growth " instead of " If there was sterile growth " in the revised manuscript. **- Describe what symptoms were considered and how the data were collected**

**Reply:** This retrospective study utilized data extracted from medical records. Urine culture was employed as the reference standard for the research. To assess the performance of Sysmex UF-5000, it was imperative to ensure that the detection outcomes from both methods were derived from the same urine sample for comparability. Based on the reviewer's suggestion, we have incorporated a flowchart in the supporting information to describe the selection of participants. 

**S1 Fig.** Flowchart of the participants included in the study

Data on pediatric patients with urine sediment and urine culture taken on the same day（n=10949）

Excluded(n=4683)

Non-first test data for the same patient

Patients remained（n=6266）

Excluded(n=1821)

Data on pediatric patients who were sent for testing on the same day but at different time

Patients with final inclusion（n=4445）

**- Add some information about where the study was conducted including some background about the setting, on the study design, prospective or** **retrospective, and on the approach for enrolling patients, alternatively whether data were obtained from medical records. Also include some data on the patient population to add with the interpretation of findings (enterococci were relatively common, Candida is usually considered a contaminant – were a considerable proportion of patients immunosuppressed)**

**Reply:** We appreciate the reviewer's comments. This retrospective study utilized data extracted from medical records.  In response to the reviewer's suggestion, we have included this detail in the methodology section. Additionally, S1 Fig displays the flowchart of participants included in the study. The interpretation of findings contents are also reflected in the discussion. The revised sections are highlighted with 'Track Changes' in red font on pages 5 and pages13-14 of the original text.

Candidauria is a common occurrence in hospitalized patients, with most patients being asymptomatic. The presence of *Candida* in urine could indicate colonization rather than an active infection in the bladder and urethra, often not necessitating treatment. The diagnostic threshold for urinary tract fungal infection is typically a fungal quantitative culture with a colony count of ≥ 10^4^/mL. Therefore, individuals suspected of having this condition should undergo quantitative fungal culture. This study examined data from children with a colony count of ≥ 10^4^/mL, these samples were considered gold standard positive when the growth of colony exceeded the gold standard definitions of a negative culture, the microorganisms isolated reflected the usual rate of uropathogens in our laboratory.

**- Data analysis: state in the text for which two parameters Sensitivity and specificity were computed**

**Reply:** The sensitivity and specificity of WBC and BACT counts were calculated at the maximum Youden index., with the optimal cut-off value being determined at the highest point of the index. To minimize false negatives, higher sensitivity levels (90%, 95%, and 99%) were proposed, resulting in corresponding cut-off values and specificities (refer to Table 2). We appreciate the feedback provided and have updated the Statistical analysis section accordingly. The revised sections are highlighted with 'Track Changes' in red font on pages 6-7 of the original text.

**3.Results
- Add percentages in the first paragraph**

**Reply:** We appreciate the reviewer's feedback and have made the required revisions in the updated version of our manuscript.The revised sections are highlighted with 'Track Changes' in red font on pages 7-8 of the original text.

**- No need to list the SE for the ROC curve AUC**

**Reply:** We appreciate the reviewer's feedback and have made the required revisions in the updated version of our manuscript. **- Explain in the methods how the “best” cut-off was selected for calculating Sensitivity and specificity**

**Reply:** Thank you for pointing this out. The largest Youden index (SE + SP-1), was utilized to determine the optimal cut-off points for distinguishing positive and negative samples. And corresponding explanations were also provided in the Statistical analysis and results section on Diagnostic performance of UF-5000.

**- Would a combination of using WBC by Sysmex and Bacterial count improve test performance.**

**Reply:** The combination of WBC and bacterial count can improve testing performance, but it is not significant, so we did not reflect it in the original text.

The specific results of the joint detection are as follows:

**- “… based on the gold standard of midstream urine cultures, the total compliance rate of UF-5000 for distinguishing UTIs is…” Please note that when considering your definitions in the methods section, not all growth represents a UTI (some may be asymptomatic bacteriuria). If necessary correct for clarity. Also please clarify what is meant by “compliance rate”**

**Reply:** BACT counts ≥ 104 CFU/mL were considered culture positive in this study. Culture positive samples were not excluded from the dataset. We appreciate the reviewer's feedback and made the necessary revisions in our manuscript. The revised sections can be identified by the 'Track Changes' in red font on page 5-6 of the original text.

**compliance rate：**A better explanation may be the consistency rate. The Consistency rate refers to the percentage of samples where the UF-5000 BACT Info marker aligns with the results of urine culture tests, out of the total number of samples. We have made the corresponding revisions in the updated version of our manuscript.

**- Explain what “unclassified flags” are**

**Reply:** In the Materials and Methods section on Sysmex UF-5000 analysis, it is explained that UF-5000 BACT-Info displays can be categorized as 'Gram-positive?' [GP], 'Gram-negative?' [GN], 'Gram Pos/Neg?' [GP/GN], or 'unclassified'. The term 'Unclassified' indicates that the UF-5000 did not detect any bacteria.

**4.Discussion**
**- The yield of urine cultures is quite low. Comment in more depth on possible reasons (in adults yield can be higher than 40% reaching 70-80%). Similarly the prevalence of Gram-negatives was relatively low (one would expect that more than 60% of samples would have Gram negative growth) – comment on possible reasons.**

**Reply:** Thank you for your feedback. In this study, positive urine culture results were low, but 10.4% in one study of children [17], aligning with our laboratory results. There may be the following reasons, and these contents are also reflected in the discussion. On the one hand, according to the health industry standard WS/T489-2016 of the People's Republic of China on 'Laboratory Diagnosis of Urinary Tract Infections', section 3 on Specimen Collection, it is recommended that patients collect morning urine and minimize water intake before bedtime. Keeping urine in the bladder for a minimum of 4 hours can help decrease the likelihood of false negative results. Children, who are less likely to retain urine for extended periods, may not provide an optimal sample as bacteria may not have sufficient time to multiply. On the other hand, diagnosing urinary tract infections in children can be challenging due to their non-specific clinical manifestations. For instance, prior antibiotic use by the child before specimen collection can lead to false negatives in traditional culture results.

Though our study population had predominantly GN bacteria, similar to other studies[2, 3, 18-20],Enterococcus is the second most common pathogen in the urinary system, accounting for 27.4%, which may be related to the fact that most of us are hospitalized patients, as many authors typically describe it as an in hospital pathogen [8,21]. The revised sections can be identified by the 'Track Changes' in red font on page 13-14 of the original text. Furthermore, the reference numbers cited align with those in the original text.

**- Replace *Enterobacteriaceae* with *Enterobacterales***

**Reply:** We greatly appreciate the reviewer’s comments. Following the reviewer’s suggestion, in the discussion, the word “*Enterobacteriaceae* “was replaced with “*Enterobacterales*”.
**- When setting cut-off values for sensitivity and specificity consider that these can also be population-dependent as well as depend on the strategy the authors decide to use for setting the cut-off (better sensitivity vs better specificity vs Youden). These might explain the slight differences between studies and there might not be a “best” cut-off in this scenario (results apply to this specific study, as you discuss in the next sentence).**

**Reply:** I appreciate all of your comments. In the results and discussion sections, we explain the state of two parameters Sensitivity and specificity were computed. The revised sections can be identified by the 'Track Changes' in red font on page 8 and page 14 of the original text.

**- “We recommend a BACT count cut-off of 7.8/μL for screening out patients with negative cultures, especially if they have no clinical symptoms of UTI.” – should these patients be tested at all if asymptomatic?**

**Reply:** Thank you for pointing this out. I have revised this section according to your suggestions. The revised sections can be identified by the 'Track Changes' in red font on page 17 of the original text. Our initial consideration was for patients monitored during treatment, where clinical doctors habitually perform both urine routine and urine culture. For this group of patients, we can suggest that if their symptoms disappear and BACT counts are less than 7.8/μL, urine culture may not be necessary.

**- Include the limitations before the conclusion**

**Reply:** We greatly appreciate the reviewer’s comments and have adjusted the order of these two parts as suggested.

**5.Tables and Figures**

**- Table 1: replace ratio with proportion, italicise species names, list in the footnote what other Gram positives and Gram negatives were isolated, also for other Fungus (other fungi); Proteus names is misspelled, consider that Candida can be a contaminant。**

**Reply:** We greatly appreciate the reviewer’s comments，According to the reviewer's suggestions, we have made corresponding modifications in Table 1, The revised sections can be identified by the 'Track Changes' in red font in Table 1 of the original text(page 9-10).

**- Table 2: add space between number and parantheses. There is typo for specimens in the first cell also for “total”**

**Reply:** Thank you for pointing this out. We have corrected these mistakes mentioned above and have carefully reviewed the spelling throughout the document.

We greatly appreciate the valuable comments made by you and reviewers. We hope that our response and revision are satisfactory and the revised manuscript is acceptable for publication.

Thank you for your consideration.

Sincerely yours,

Xin Lv: Ph.D., M.D.

Clinical Laboratory, Children's Hospital Affiliated to Shandong University, 23976 Jing-Shi Road, Jinan 250022, Shandong Province, PR China;

Email: etyyjyklvxin@163.com

Tel: +8618866110936
